# Distinctness of Electroluminescence and Optical Gain in Laser Diodes with Wide Polar Quantum Wells

**DOI:** 10.1021/acsphotonics.4c02193

**Published:** 2025-03-06

**Authors:** Mateusz Hajdel, Krzysztof Gołyga, Marcin Siekacz, Anna Feduniewicz-Żmuda, Czesław Skierbiszewski, Ulrich Theodor Schwarz, Grzegorz Muziol

**Affiliations:** † Institute of High Pressure Physics Polish Academy of Sciences, Sokolowska 29/37, 01-142 Warsaw, Poland; ‡ Institute of Physics, Chemnitz University of Technology, Reichenhainer Str. 70, 09126 Chemnitz, Germany

**Keywords:** InGaN, GaN, quantum-confined Stark effect, spontaneous emission, amplified spontaneous emission

## Abstract

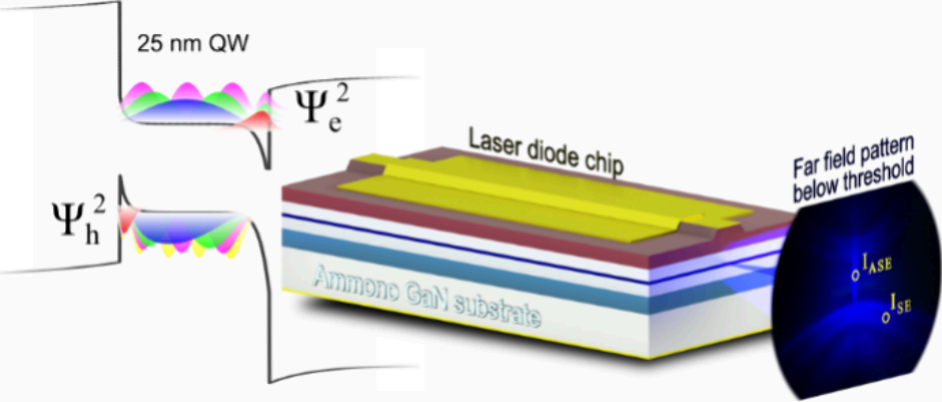

Despite the ubiquity of semiconductor-based emitters in optoelectronic devices we use every day, obstacles still remain to unlock their full potential. One of these lies in long-wavelength GaN-based laser diodes (LDs). It is common knowledge that InGaN quantum wells (QWs) exhibit extremely large built-in polarization, which helps to obtain long-wavelength emission in light-emitting diodes, thanks to the large quantum-confined Stark effect. However, in this paper, it is shown that in order to achieve long-wavelength LDs, wide InGaN QWs might be preferential. The lasing wavelength for blue LDs can be even 20 nm longer in the case of wide QWs than in thin QWs for the same composition. The mechanisms behind these effects are explored by analyzing evolution of spontaneous emission, amplified spontaneous emission, optical gain, and quasi-Fermi level separation. It is shown that in wide QWs, the spontaneous emission originates from highly excited states. However, as the carrier density increases, quantum states with lower energy take over. Furthermore, population inversion, and thus lasing action, is obtained from the lowest excited states, resulting in long-wavelength lasing. The reported effects should also be observed in other polar materials with sufficiently thick QWs.

## Introduction

GaN-based light-emitting diodes (LEDs), thanks to their high efficiency, have taken the leading position as artificial light sources. They are widely spread in the market of general lighting and also more and more commonly used in a wide range of self-emissive displays.^1−4^ For other applications like communication systems,^5−9^ light projection,^10−12^ or near-absolute zero cooling systems,^13^ laser diodes (LDs) are the best candidates to meet the set requirements. The wide range of application possibilities and enormous potential of the GaN material system for realizing reliable and compact devices pushed many research groups in the world to study III-nitride compounds. In state-of-the-art III-nitride light emitters, the active region is built with multiple InGaN quantum wells (QWs) with thicknesses in the range of 2–4 nm.^14^ The presence of the built-in piezoelectric field and the resulting quantum-confined Stark effect (QCSE) in this material system significantly alter the confinement energy and emission wavelength.^15−17^ Additionally, the spatial separation of electron and hole wave functions leads to decreased oscillator strength.^18^ These effects are more prominent for layers with high indium content, where the built-in fields are larger.^19^ From the perspective of realizing long-wavelength LDs and the integration of all red–green–blue (RGB) emitters on a single InGaN platform, this effect is highly undesirable. On the other hand, recent works show that III-nitride LEDs and LDs with a single-wide InGaN QW (with thickness above 10 nm) can have higher efficiencies when compared to devices with thin QWs (with thickness below 4 nm).^20−25^ Significant effort has been made to understand operation mechanisms of the wide InGaN QWs.^23,26−35^ It was shown that upon excitation, the injected carriers, instead of recombining, accumulate on the opposite interfaces of the QW. This leads to screening of the built-in field and rearrangement of carrier wave functions. Screening ends when highly efficient recombination paths through excited states start to dominate. Interestingly, the fraction of carriers which are in the ground states is still localized close to the edges of the QW. It was shown experimentally that without excitation, the built-in field is present even in the 25 nm wide QW^36^ and is completely screened under excitation.^37^

In this work, we simultaneously study the spontaneous emission (*I*_SE_), amplified spontaneous emission (*I*_ASE_), optical gain, and quasi-Fermi level separation in both thin and wide InGaN QWs. The optical gain of LDs with 2.6, 10.4, and 25 nm QW is extracted from *I*_SE_ and *I*_ASE_ spectra^38−42^ and compared with optical gain measured with the use of the Hakki–Paoli method.^43,44^ We show that there exists a difference in the origin of the optical gain and spontaneous emission in wide InGaN QWs. At low excitation, spontaneous emission arises from the recombination of carriers occupying high-energy excited states; however, as the excitation is increased, excited states with lower indexes start to dominate the recombination. At the same time, the optical gain is observed only for lowest-energy excited states. This adds a new layer of understanding to the mechanism of light emission from the excited states in wide InGaN QWs.

## Results and Discussion

**Figure 1:**
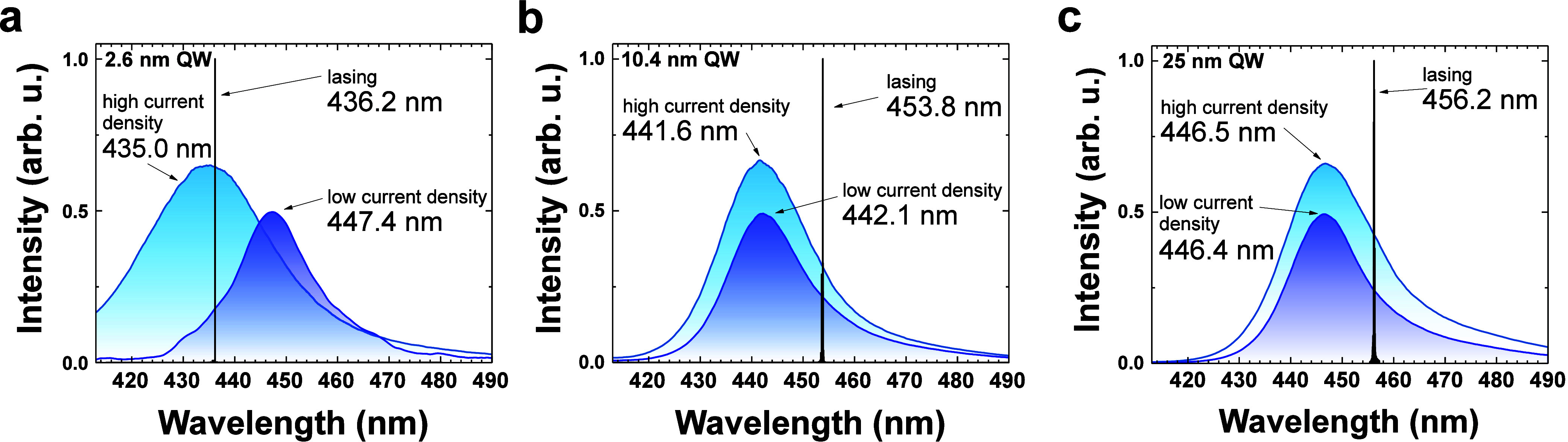
Comparison of the electroluminescence spectra measured on as-grown wafer at a low current density of 20 A/cm^2^ and on processed laser chips just below and just above lasing thresholds for samples with QW thicknesses of (a) 2.6, (b) 10.4, and (c) 25 nm.

First, we compare three electroluminescence spectra for each LD in Figure 1. The first spectrum is pure spontaneous emission collected from the as-grown wafers at a low current density of 20 A/cm^2^. The remaining two spectra were measured on processed laser chips. The second spectrum was collected at a high current density just below the lasing threshold. The third spectrum shows lasing just above the threshold. In the case of LDs with 2.6 nm QWs, the as-grown wafer emits with a maximum at λ = 447 nm, while for higher current, the peak shifts to λ = 435 nm, and lasing occurs at λ = 436 nm. The observed shift of emission is due to the screening of the built-in piezoelectric field.^15,16,45,46^ For LDs with a wide QW, the emission from the as-grown wafer is at a slightly shorter wavelength when compared to the thin QW case. The emission maximum is at λ = 442 and 446 nm for the 10.4 nm QW and 25 nm QW, respectively. Interestingly, there is a stunning difference in the spontaneous emission at a high current density and in the lasing wavelength. The spontaneous emission does not shift with current density, while the lasing emission is red-shifted with respect to spontaneous emission by about 10 nm for both cases. The red shift of laser emission with respect to spectrum collected below the threshold was never observed in GaN-based LDs but is very similar to the behavior observed in LDs operating in the infrared spectrum regime.^47−52^

**Figure 2:**
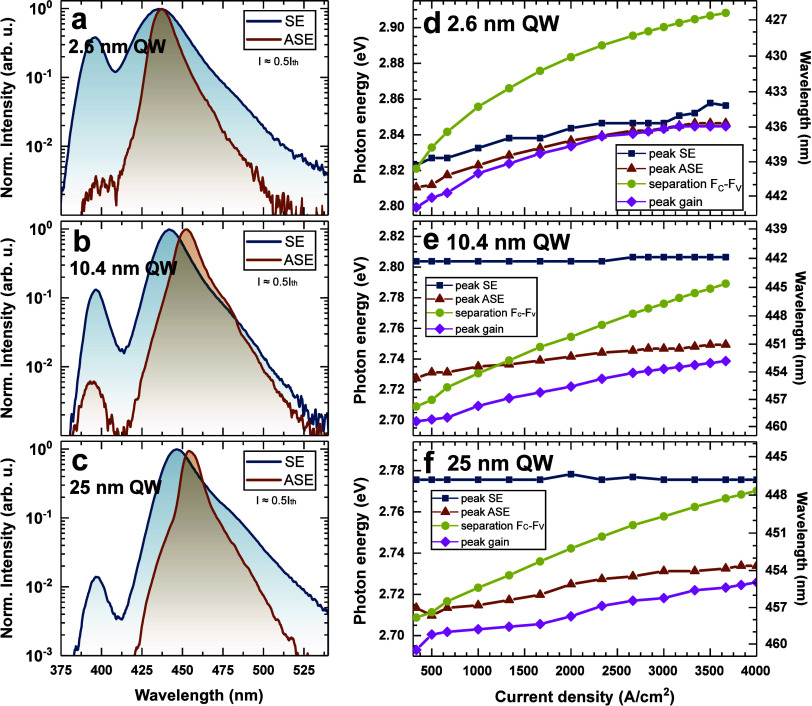
Exemplary measurements of *I*_SE_ and *I*_ASE_ at I ≈0.5*I*_th_ for LDs with the QW thicknesses of (a) 2.6, (b) 10.4, and (c) 25 nm. Dependence of spontaneous emission, amplified spontaneous emission, optical gain, and quasi-Fermi level separation on current density for LDs with the QW thicknesses of (d) 2.6, (e) 10.4, and (f) 25 nm.

To explain this observation, we studied the evolution of *I*_SE_ and *I*_ASE_ with current from LD chips below the threshold. In Figure 2a–c, exemplary *I*_SE_ and *I*_ASE_ spectra are presented for about half of the threshold current. In the case of *I*_SE_, two peaks can be observed. The main one is the emission from the QW, while the second peak at 400 nm is due to recombination from the InGaN waveguide between the QW and electron blocking layer. The intensity of the peak from the waveguide decreases with the increase of the QW width. Two mechanisms can be responsible for such a behavior. First, the electron capture can be increased for wider QWs. Second, the carrier escape for wide QWs can be lower due to the lower confinement energy. Nevertheless, the wide QW seems to prevent electron overflow. In the case of *I*_ASE_, the intensity of the peak from the waveguide is greatly decreased. This happens because the light of high energy propagating in the waveguide is absorbed by the QW. Figure 2d–f presents the evolution of *I*_SE_ and *I*_ASE_ maxima in the whole measured current density range. For the LD with 2.6 nm QWs, both *I*_SE_ and *I*_ASE_ shift to higher energies. This is well-known and is explained by screening of the QCSE.^46^ A small difference of 10 meV between the maxima of *I*_SE_ and *I*_ASE_ is observed in almost the whole current density regime. This implies that the same carriers are responsible for the spontaneous and stimulated emission. Interestingly, for the wide QWs (10.4 and 25 nm), the position of *I*_SE_ is independent of current in the whole measured range, which was observed and reported by us before.^21−23,26^ On the other hand, *I*_ASE_ occurs at a much lower energy and blue shifts with current. The difference between *I*_SE_ and *I*_ASE_ is 75 and 65 meV at the lowest current and becomes 56 and 44 meV near the threshold for 10.4 and 25 nm QWs, respectively. The wavelength stability of *I*_SE_ and the change in emission wavelength of *I*_ASE_ will be investigated further using modeling after description of the rest of the data.

**Figure 3:**
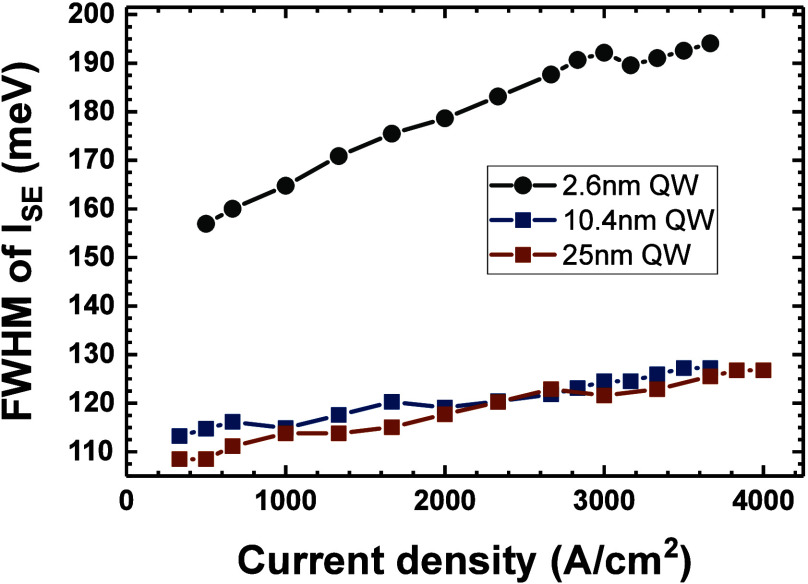
Dependence of full width at half-maximum of the spontaneous emission on current density for LDs with 2.6, 10.4, and 25 nm QWs.

It is interesting to compare the spectral broadening of the spontaneous emission between thin and wide QWs. The inhomogeneous broadening in InGaN QWs has been widely studied both experimentally and theoretically.^53−56^ However, in the case of thin InGaN QWs, it is difficult to experimentally distinguish the effects of inhomogeneities of thickness and composition of the QW on broadening of photoluminescence. This problem is further enhanced by the fact that inhomogeneities in composition change not only the band gap but also the local polarization, while inhomogeneities in thickness strongly affect the QCSE. Here, we can study broadening of photoluminescence only due to band gap change with composition because in the case of the wide QWs, the polarization field is close to being fully screened, while the effects of thickness fluctuations on the scales of monolayers (MLs) are negligible. The full width at half-maximum (FWHM) of *I*_SE_ for studied LD devices is shown in Figure 3. The FWHM is equal to 157 and 110 meV in the case of thin and wide QWs, respectively. Jarema et al. calculated separately the influence of inhomogeneities of thickness and composition of polar and nonpolar InGaN QWs on broadening of photoluminescence.^56^ The nonpolar QW case can be used to resemble our wide QWs because the electric field is mostly screened. The calculated indium fluctuations (Δ*x*), which would lead to a broadening of 110 meV, similar to the observed in wide InGaN QWs, are equal to Δ*x* = 0.01.^56^ In the case of polar QWs, the sole effect of composition fluctuations of Δ*x* = 0.01 would result in a FWHM of only 120 meV, which is lower than the experimentally measured value. Therefore, we can see that thickness fluctuation also plays a role in thin QWs. Fortunately, Jarema et al. also calculated the combined effect of both thickness (Δ*d*) and composition fluctuation. It was found that a FWHM of 157 meV can be expected for Δ*x* = 0.01 and Δ*d* = 1 ML.^56^ We can therefore conclude that in the studied QWs, the fluctuations of composition and thickness are on the order of Δ*x* = 0.01 and Δ*d* = 1 ML, respectively.

**Figure 4:**
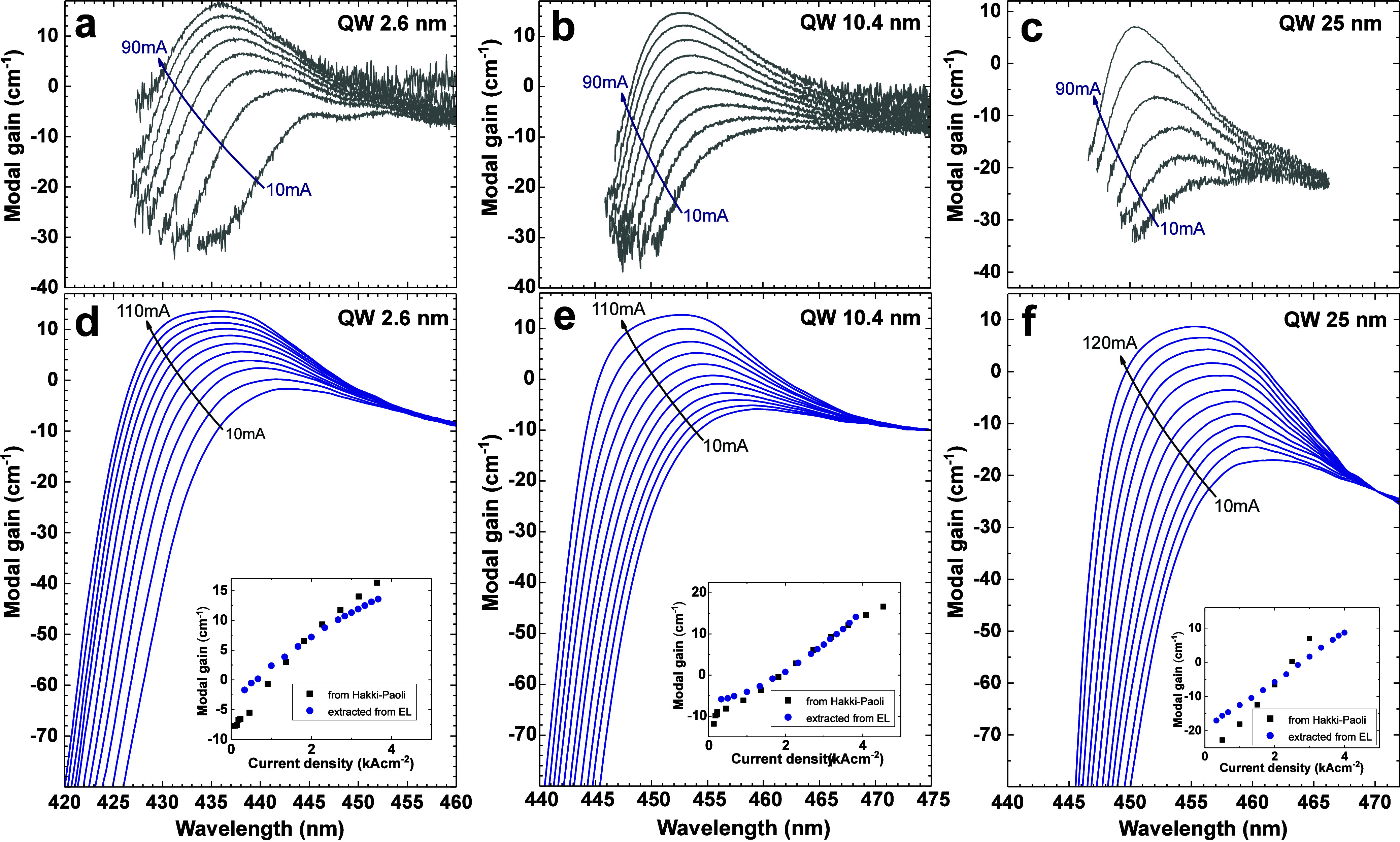
Modal gain spectra measured with the Hakki–Paoli method for the LDs with (a) 2.6, (b) 10.4, and (c) 25 nm wide QWs, respectively. (d–f) Gain spectra extracted from *I*_SE_ and *I*_ASE_ for the same QW thicknesses. Insets show the dependence of maximum modal gain on the current density extracted at the peak of each gain spectrum.

We extracted the gain spectra using the *I*_SE_ and *I*_ASE_ measurements for driving currents from 10 up to 110 mA for 2.6 and 10.4 nm QWs and up to 120 mA for LDs with 25 nm QWs. In Figure 4, we compare them with the gain spectra measured by the Hakki–Paoli method measured in the 10–90 mA current range. The data are taken from different chips from the same epitaxial process in the case of 2.6 and 10.4 nm QWs. In the case of LDs with 25 nm QWs, a sample from a duplicate epitaxial growth was used. Despite analyzing different chips, the overall tendency and the position of the gain maximum peaks are comparable between the two methods. For the currents near the lasing threshold, the *I*_ASE_ peak becomes more and more narrow, and eventually, the resolution of the used spectrometer is not sufficient to properly resolve the shape of the peak. As a result, the flattening of the high current gain spectra is visible in Figure 4d–f. It is worth noticing that the gain extracted from *I*_SE_ and *I*_ASE_ data can be determined for much shorter wavelengths than in the case of the Hakki–Paoli method. This is due to the limitations of the Hakki–Paoli method because the cavity-modulated peak–valley signals are not well resolved.^57^

In order to obtain insight into the nature of optical transitions in the wide QWs, we determined the quasi-Fermi level separation (QFLS). To extract the QFLS from optical gain, we first estimated the value of modal gain without excitation by linear approximation of data from the inset to Figure 4. The energy at which the gain drops to this level corresponds to energy above which the gain of the QW changes to absorption, i.e., QFLS.^58^ It is worth mentioning that the method of determination of the QFLS is independent of the level of internal losses used for the calculation of the gain spectra from electroluminescence data since they only affect the gain value and not the energy. The extracted values of QFLS are plotted in Figure 2d–f marked as *F*_c_–*F*_v_. For the wide QW cases, we can observe that spontaneously emitted photons originate from states which are above the QFLS. It is important to note that the QFLS is always below the *I*_SE_ for the wide QWs. It shows that the spontaneous emission comes mainly from carriers, which do not exhibit population inversion. This observation leads to important implications on the understanding of the emission mechanisms in the wide QW LDs.

**Figure 5:**
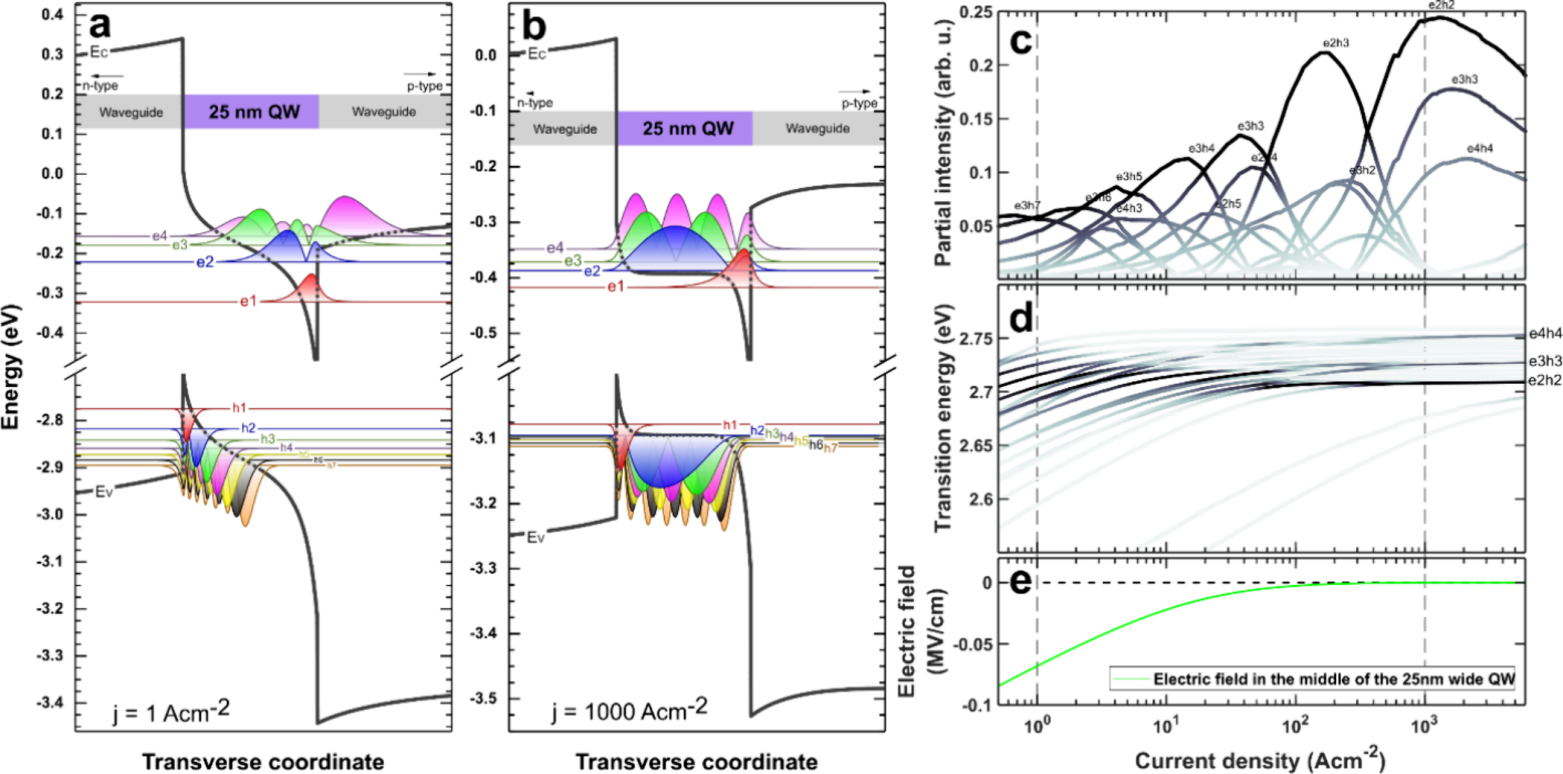
Simulated band structure of a 25 nm wide QW at (a) *j* = 1 A cm^–2^ and (b) *j* = 1000 A cm^–2^ with wave functions of the first seven heavy hole and four electron levels. Energy values are indicated by base lines, and wave functions are normalized and scaled for better visibility. (c) Calculated dependence of partial intensity of the most important transitions on current density. (d) Dependence of transition energies for transitions presented in (c) on current density. The color scale represents the contribution to total intensity and is identical to that in panel (c). (e) Dependence of the electric field in the middle of the 25 nm wide QW on current density. The dashed vertical lines in panels (c), (d), and (e) indicate the excitation levels, which correspond to band structures shown in panels (a) and (b).

Studying the mechanism responsible for the emission wavelength stability of spontaneous emission and the contribution of the exact states to the recombination process, we performed drift–diffusion simulations, followed by solving the Schrodinger equation for a wide QW LD structure using SiLENSe 6.4. The first four electron and seven heavy hole states are shown in Figure 5 for the 25 nm wide QW in (a) low (*j* = 1 A cm^–2^) and (b) high (*j* = 1000 A cm^–2^) excitation conditions. At low excitation, there is a non-negligible electric field in the QW, and it separates carriers occupying the ground and excited states. Therefore, only the excited states, whose wave functions penetrate to the middle of the QW, exhibit substantial overlap. In this particular case, the lowest energy transition with a non-negligible wave function overlap comes from the e3h6 transition. At higher excitation, the increased number of carriers screens the built-in field, leading to an almost flat band condition in the center part of the well. Most interestingly, the wave function overlap between excited states with a lower index, such as e2h2, becomes significant. Only the ground states remain spatially separated because they are confined in small potential minima on each interface of the QW. The e1h1 wave function overlap is below 1 × 10^–5^, which implies that the ground states remain dark no matter the excitation of the wide QW. In Figure 5c, the dependence of partial contribution to the emission of the most important transitions on current density is shown. At low current density, transitions involving very high electron and hole states contribute the most to the emission. Surprisingly, as the current injection increases, the transitions involving states with lower indexes start to contribute the most. This drop of indexes ends on the e2h2 transition, which has the highest overlap at the high current density.

This is a stunning manifestation of how unusual the behavior of uniaxial heterostructures can be. The described mechanism has an important impact on the emission energy and partially explains the observed emission wavelength stability of *I*_SE_ for structures with wide QWs. In Figure 5d, the energy of transitions is shown with the color intensity corresponding to the partial contribution to the emission intensity from Figure 5c. The dependence of the electric field in the middle of the QW on current density is shown in Figure 5e. As the current density increases, the electric field is being screened, and the energy of the states and corresponding transitions increase. However, at the same time, the radiative emission occurs through lower and lower states, striving to e2h2. As a result, the emission energy remains fairly constant, until the lowest possible transition is reached. This coincides with full screening of the electric field in the middle of the QW. Then, as the carrier population still increases, the energies of states remain fairly constant; however, the emission energy should shift toward higher energies due to band filling.^58,59^ In Figure 3, we observed the increase of FWHM with increased excitation for all samples, which suggests that the band filling effect occurs in both thin and wide QWs. The reason why the change of the emission wavelength in the high currents is not observed experimentally in wide QWs may be due to the band gap renormalization. The many-body interactions in highly excited semiconductors can lead to the screening of the crystal field by carriers, and a shrinkage of the band gap can be observed. Previous works show that in GaN, band gap renormalization strongly depends on the density of electron–hole plasma.^60^ The band filling effect and the decrease of band gap due to renormalization compete each other, and as a result, no shift in emission can be observed as the excitation level increases.^61,62^ This is supported by our experimental observation of the wavelength stability of the wide QW samples for the very high current density regime.

**Figure 6:**
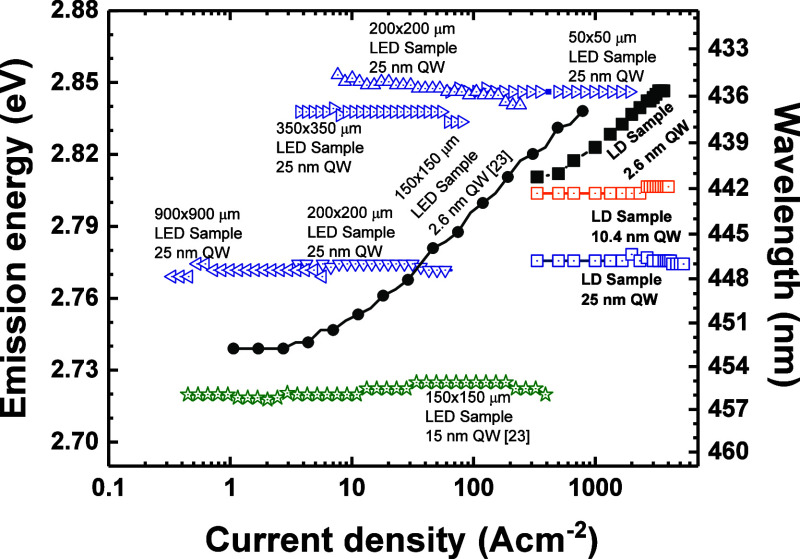
Dependence of spontaneous emission energy on the current density. Solid points represent data from devices with a 2.6 nm QW. Hollow points are from different LED and LD samples with wide QWs.

In Figure 6, we collected data from different epitaxial processes of the LED and LD samples with thin and wide QWs. The prevalence of the wavelength stability phenomenon in wide QW samples, as well as the range of current densities where it can be observed, is astonishing. The difference in emission wavelengths among samples comes from the fact that they are from several epitaxial processes, and differences in In composition are present, which additionally confirms the omnipresence of the wavelength stability phenomenon in wide QWs. The LED samples are driven with direct current; therefore, a small red shift at the highest excitations due to heating is visible.

If the band gap renormalization equalizes the band filling in the studied current density regime, the same should also apply for thin QWs. Then, the blue shift in case of thin QWs should originate only due to screening of the QCSE. This agrees with studies performed on superluminescent diodes.^46^ Interestingly, even at the highest current densities, at which heating, band filling, and band gap renormalization start to play a role, the carrier density is still insufficient to fully screen the polarization field.^46^ It was also shown that for extremely high excitation of over 300 kA cm^–2^ possible in micro LEDs, eventually a redshift of emission can be observed and is attributed to the band gap renormalization.^63^

## Conclusions

In this work, aspects of emission from the wide QW LD based on the polar material were studied in a broad perspective. We have found a significant difference in the lasing wavelength of the InGaN QW with various thicknesses but with the same In content of 17%. By increasing only the thickness of the QW, the achieved lasing wavelength can be even 20 nm longer. Therefore, if the problem with strain relaxation can be mitigated, the wide QWs can be very beneficial for realizing the green- and red-emitting lasers in the GaN material system. Furthermore, we have shown that the steady spontaneous emission energy of devices with wide InGaN QWs is not proof that the built-in electric field is fully screened. Instead, at low excitation density, the field is not fully screened, and the emission originates from highly excited states. As the excitation level is increased, the added carriers lead to further screening of the built-in field. Unexpectedly, this promotes lower excited states to contribute more to the emission. In other words, the wide InGaN QWs constitute a unique system, in which the index of quantum states responsible for emission decreases with excitation. Furthermore, we have shown that in the case of LDs with wide QWs, the optical gain originates from quantum states with energy lower than states generating the spontaneous emission. In fact, we showed that the quasi-Fermi level separation does not surpass the spontaneous emission energy, which means that the carriers contributing to spontaneous emission do not even experience population inversion. The results reported here broaden the understanding of light generation processes in LDs with wide polar QWs.

## Materials and Methods

**Figure 7:**
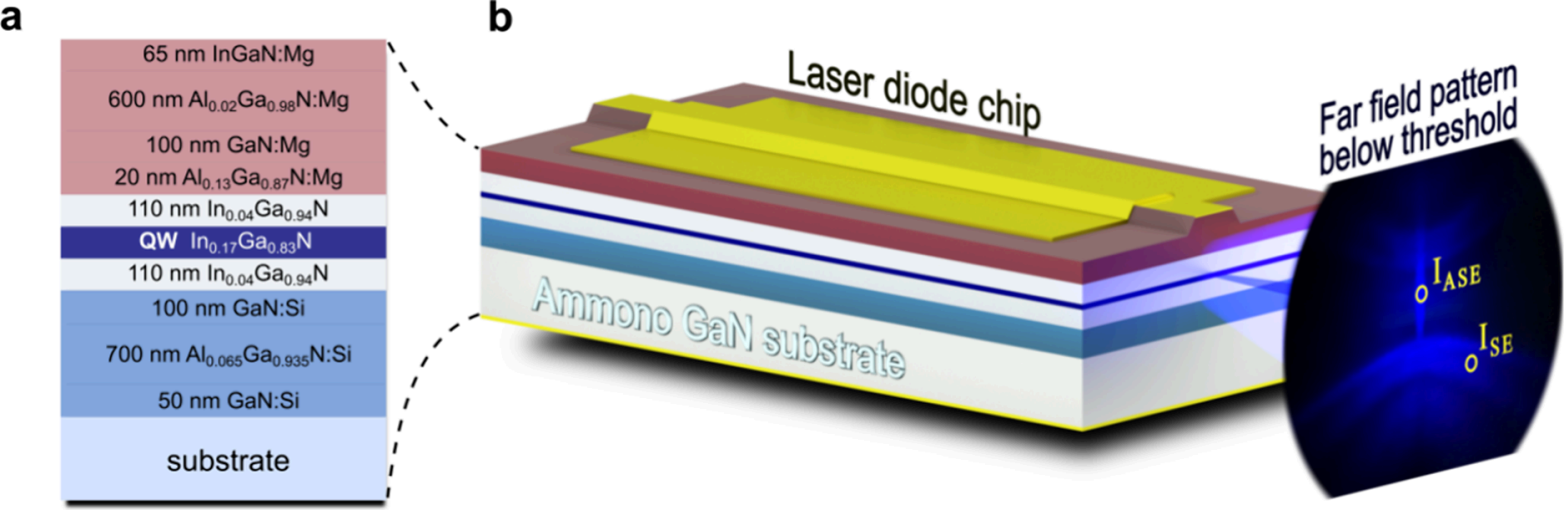
(a) Schematic structure of measured laser diodes. (b) Laser diode chip and pattern emitted below the lasing threshold with two areas, in which spontaneous emission *I*_SE_ and amplified spontaneous emission *I*_ASE_ spectra were collected.

The LD structures were grown using plasma-assisted molecular beam epitaxy on bulk Ammono-GaN freestanding substrates with a threading dislocation density on the order of 1 × 10^4^ cm^–2^.^64^ The LD structures were grown starting with 50 nm GaN:Si, 700 nm Al_0.065_Ga_0.935_N:Si cladding, and 100 nm GaN:Si, each with a Si concentration of 2 × 10^18^ cm^–3^. Next, a 220 nm In_0.04_Ga_0.96_N waveguide was grown with either a 2.6, 10.4, or 25 nm In_0.17_Ga_0.83_N single QW located in the center. After that, a 20 nm Al_0.13_Ga_0.87_N:Mg electron blocking layer is placed with a Mg concentration of 3 × 10^19^ cm^–3^. The AlGaN electron blocking layer was grown at the same temperature as the InGaN QW and InGaN waveguide with no interruption of the growth process. The change in the material was achieved by opening and/or closing shutters for aluminum, gallium, and indium effusion cells. The *p*-type cladding starts with 100 nm GaN:Mg, followed by 600 nm Al_0.02_Ga_0.98_N:Mg. Both layers have a 1 × 10^18^ cm^–3^ Mg concentration. The structure is capped with a 65 nm InGaN:Mg contact layer. Schematic of the epitaxial structure is presented in Figure 7a. More details of the growth of LD structure were published in previous work.^65^

First, electroluminescence spectra were collected from as-grown wafers by using indium contacts. Next, the samples were processed into standard edge-emitting laser chips with a 3 μm ridge width and a 1000 μm resonator length with Ti/Al/Ni/Au and Ni/Au metallization for the *n*-type and *p*-type, respectively. The mirror facets were left uncoated. The emitted far-field pattern below the lasing threshold of an exemplary chip is shown in Figure 7b. A narrow vertical pattern can be distinguished in the middle. It consists solely of light that was propagating inside the waveguide. The rest of the emitted light, which was not coupled into the waveguide, is extracted at various angles from the LD chip. By placing an optical fiber in the vertical pattern, we were able to collect the amplified spontaneous emission (*I*_ASE_). Moving the optical fiber to the side allowed for the measurement of the spontaneous emission (*I*_SE_) spectra from the same LD chip. The spectra were measured with an Ocean Optics USB4000 spectrometer. It is important to note that this technique allows us to measure both *I*_ASE_ and *I*_SE_ independently in a wide excitation regime, from very low current densities up to a threshold current density.

We will now describe how the optical gain is derived from *I*_SE_ and *I*_ASE_. Let us consider an amplifying medium, where photons are both generated and amplified. The light intensity coming out of such a medium of length *L* is given by^40^
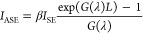
1where *G*(λ) is the modal gain and β is a geometrical factor describing the part of photons, which are coupled into the medium and contribute to the *I*_ASE_. Additionally, in the case of LDs, the presence of cavity needs to be included. The consequent reflections of the light from the resonator mirrors influence the light intensity leaving the cavity. The expression for *I*_ASE_ extracted through mirror *R*_1_ can be written as^66,67^
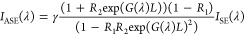
2where *R*_1_ and *R*_2_ are the mirror reflectivities and the *I*_SE_ (λ) and *I*_ASE_ (λ) are the measured values of intensity from Figure 7. The bottleneck of this method is that *I*_SE_ in eqs 1 and 2 refers to the whole light emitted from the QW. Unfortunately, it cannot be directly measured, as it is partially absorbed by the metal contacts. Therefore, we introduce a scaling factor γ, which is the product of the collection efficiency and the spontaneous emission factor β. The modal gain is defined by material gain *g*, confinement factor Γ of optical mode with the active region, and optical losses *a*_*i*_ through the relation: *G*(λ) = Γ_*g*_(λ)–*a*_*i*_. When the modal gain value is known for a certain wavelength, one can calculate the value of γ, and then, the whole gain spectra can be reproduced from eq 2. We calculated γ from *G* measured by the Hakki–Paoli method in the long-wavelength regime, in which *g* = 0 and the modal gain equals the internal losses. Then, we used the obtained γ value to numerically solve eq 2 for *G*(λ). Alternatively, the level of internal losses could also be measured by the variable stripe method. In order to verify the gain spectra extracted using eq 2, we compare it to G measured using the Hakki–Paoli method,^43,44^ with an FHR1000 Horiba Jobin Yvon 1-m-long spectrometer equipped with a 3600 mm^–1^ grating and a CCD camera. It is important to note that the Hakki–Paoli gain spectra do not resolve the high energetic part of the spectrum, especially for high current excitation; thus, for the determination of the quasi-Fermi level separation, the gain calculated from *I*_SE_ and *I*_ASE_ was used.

## Supporting Information Available

Simulation result for the device with a single 2.6 nm QW (complementary to Figure 5) (Figure S1) (PDF)
Animation of the wave functions of electron and hole states in a 25 nm wide 17% InGaN quantum well for increasing current density (extension of Figure 5a,b) (MP4)
