# Supplementary material for: Distinctness of Electroluminescence and Optical Gain in Laser Diodes with Wide Polar Quantum Wells
Source: ACS Photonics. 2025 Mar 6;12(3):1515–23. doi: 10.1021/acsphotonics.4c02193 (PMC11926953; doi:10.1021/acsphotonics.4c02193)
Supplement: Supplementary file 1 — ph4c02193_si_001.pdf [file ph4c02193_si_001.pdf]

Supporting Information: Distinctness of Electroluminescence and Optical Gain in Laser Diodes with Wide Polar Quantum Wells

Mateusz Hajdel, Krzysztof Gołyga, Marcin Siekacz, Anna Feduniewicz-Żmuda, Czesław Skierbiszewski, Ulrich Theodor Schwarz and Grzegorz Muziol

This Supporting Information document is 1 page long and includes 1 figure:

Figure S1: Simulation result for device with single 2.6 nm QW. Complementary to Figure 5.

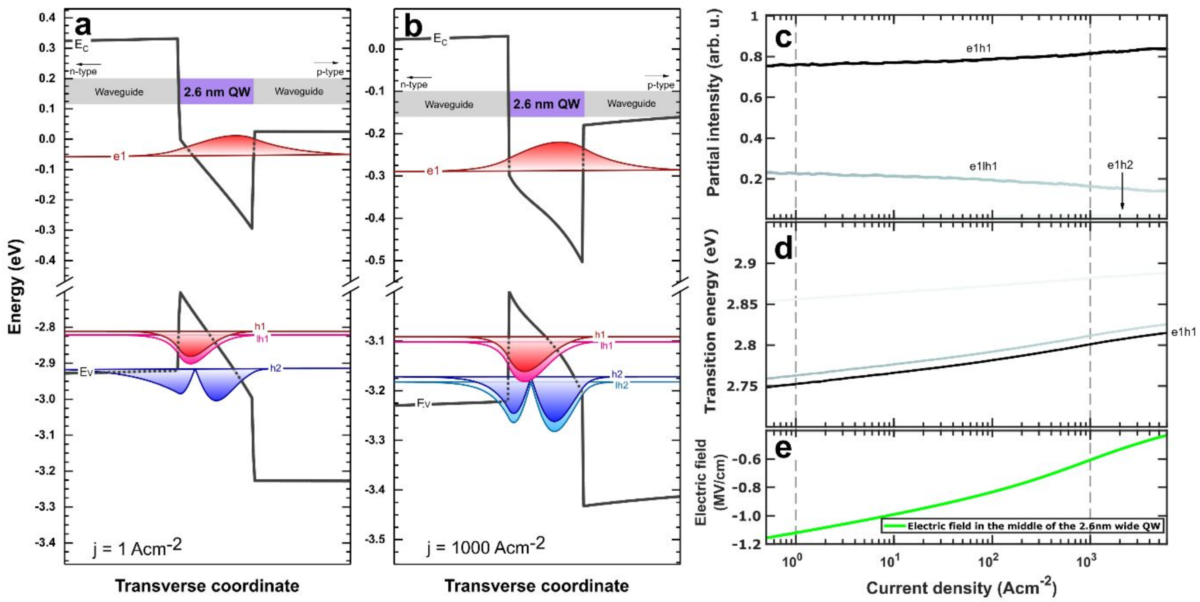

Figure S1. Simulated band structure of a 2.6 nm QW at a) j = 1 Acm^{-2} and b) j = 1000 Acm^{-2} with wavefunctions of the heavy, light holes and electron level. Energy values are indicated by base lines, and wavefunctions are normalized and scaled for better visibility. c) Calculated dependence of partial intensity of the most important transitions on current density. d) Dependence of transition energies for transitions presented in c) on current density. The color scale represents the contribution to total intensity and is identical as in c). e) Dependence of electric field in the middle of the 2.6 nm QW on current density. The dashed vertical lines in c), d) and e) indicate the excitation levels which correspond to band structures shown in a) and b).
